# Cryopreservation of primary cultures of mammalian somatic cells in 96-well plates benefits from control of ice nucleation

**DOI:** 10.1016/j.cryobiol.2020.02.008

**Published:** 2020-04

**Authors:** Martin I. Daily, Thomas F. Whale, Riitta Partanen, Alexander D. Harrison, Peter Kilbride, Stephen Lamb, G. John Morris, Helen M. Picton, Benjamin J. Murray

**Affiliations:** a Institute of Climate and Atmospheric Science, School of Earth and Environment, University of Leeds, Leeds, LS2 9JT, UK; b Discovery and Translational Science Department, Leeds Institute of Cardiovascular and Metabolic Medicine, School of Medicine, University of Leeds, Leeds, LS2 9JT, UK; c Asymptote Ltd (GE Healthcare), Sovereign House, Cambridge, CB24 9BZ, UK

## Abstract

Cryopreservation of mammalian cells has to date typically been conducted in cryovials, but there are applications where cryopreservation of primary cells in multiwell plates would be advantageous. However excessive supercooling in the small volumes of liquid in each well of the multiwell plates is inevitable without intervention and tends to result in high and variable cell mortality. Here, we describe a technique for cryopreservation of adhered primary bovine granulosa cells in 96-well plates by controlled rate freezing using controlled ice nucleation. Inducing ice nucleation at warm supercooled temperatures (less than 5 °C below the melting point) during cryopreservation using a manual seeding technique significantly improved post-thaw recovery from 29.6% (SD = 8.3%) where nucleation was left uncontrolled to 57.7% (9.3%) when averaged over 8 replicate cultures (*p* < 0.001). Detachment of thawed cells was qualitatively observed to be more prevalent in wells which did not have ice nucleation control which suggests cryopreserved cell monolayer detachment may be a consequence of deep supercooling. Using an infra-red thermography technique we showed that many aliquots of cryoprotectant solution in 96-well plates can supercool to temperatures below −20 °C when nucleation is not controlled, and also that the freezing temperatures observed are highly variable despite stringent attempts to remove contaminants acting as nucleation sites. We conclude that successful cryopreservation of cells in 96-well plates, or any small volume format, requires control of ice nucleation.

## 1 Introduction

A range of cells are routinely cryopreserved in millilitre volumes of aqueous cell suspensions, but successfully cryopreserving cells using a controlled rate freezing method in smaller volumes such as those used in 96-well microplates is, in comparison, more challenging. This is in part because small volumes of water and aqueous solutions tend to supercool by many degrees below the melting point before the onset of ice-nucleation and subsequent ice growth [18,37]. It is well established that inducing extracellular ice formation at relatively warm supercooled temperatures during controlled rate freezing is beneficial to the post-thaw viability of many cell types [22,23,25,34,36,43]. However this is still a significant uncontrolled variable during many cell and tissue cryopreservation procedures [30].

The efficacy of controlled rate freezing is often qualitatively understood by the two-factor hypothesis [28]. This hypothesis states that cooling must be sufficiently slow for cells to dehydrate and avoid intracellular ice formation, but quick enough to avoid other detrimental impacts of low temperature on cells. When ice nucleation is controlled (i.e. extracellular ice crystals are actively nucleated) during cryopreservation, pure water migrates to and is locked away in ice crystals, resulting in the inter-crystal channels where cells reside becoming increasingly concentrated in solutes. This osmotic imbalance drives cellular dehydration [26]. Dehydration reduces the water activity of intracellular solutions which lowers the freezing point of the cytoplasm and thus favours solidification of the cytoplasm in a survivable, non-crystalline state [11,27,29]. In contrast, without controlled extracellular ice formation, cells dehydrate less, increasing the chance of intracellular ice formation, which is usually fatal [24].

Another factor to consider is the sudden rise in temperature resulting in a ‘thermal shock’ from the release of latent heat of crystallisation followed by a rapid cooling as the system comes back to equilibrium with its surroundings. The magnitude of this thermal shock increases with the depth of supercooling. Finally, there is evidence that following ice nucleation at warm supercooled temperatures cell membranes of mammalian somatic cell lines [3,43] and horse spermatozoa [33] undergo a dehydration induced phase transition, which is absent when ice forms at colder temperatures and it is possible that this lyotropic phase transition is beneficial for cell survival. In earlier work on the topic samples were seeded with ice at warm supercooled temperatures to ensure crystallisation [28]. However the practice of controlling ice nucleation is to date prevalent only in a few areas, notably oocyte and embryo cryopreservation for reproductive biology where failure to control ice nucleation is clearly detrimental [15].

While the cryovial is the standard vessel used for the shipping of cryopreserved cells and for the long-term storage of complex human tissues with clinical therapeutic application the routine cryopreservation of mammalian cells in 96-well culture plates would be beneficial as many somatic cell bioassays require this format. Ice nucleation, however, tends to occur both at lower temperatures and with greater variability within the sub-millilitre volumes of liquid used in multiwell plates than in millilitre volume vials [30]. This poses the problem of both poor and variable post-thaw cell viability which would be an unacceptable baseline condition in plates used, for example, for cytotoxicity assays. A great deal of standard analytical equipment is built around the multiwell plate format allowing, for example, standard *in-vitro* pharmacological experiments to be rapidly performed on large numbers of samples. Specifically, routine cryopreservation in 96-well plates would facilitate ADME (adsorption, distribution, metabolism, excretion) and toxicology screening of drug compounds against new drug molecules by allowing large, homogeneous batches of relevant cell types to be frozen and shipped to toxicology labs and stored for use over extended time periods. Despite these advantages commercial suppliers of cells for scientific research may at present offer only plated cells shipped fresh or cryopreserved cells shipped in cryovial format for subsequent thawing and seeding into plates [31]. The former format raises logistical challenges resulting in high shipping costs while the thawing and re-plating associated with the latter is inefficient and incurs additional time and labour. Overall both these delivery formats increase the cost and reduce the effectiveness of toxicology assays for the end user.

Whilst not currently available ‘off the shelf’, demonstrations of cell cryopreservation in microplated monolayer format do exist in the literature. Protocols have been devised for freezing of immortalised cell lines [[7], [8], [9],^16^,^19^], embryonic stem cells [32] and hybridomas [41]. A frequently observed issue when attempting to freeze cells in this is way is post-thaw detachment of cells from the substrate. Campbell et al. [10] devised a controlled warming methodology upon thawing to prevent cell detachment, attributing this phenomenon to thermal expansion stresses within the plate upon rapid warming. The use of immortalised and previously cryopreserved cell lines means that many studies are conducted with cells that are relatively resistant to the damage caused by cryopreservation. This may distort the true effect of ice nucleation control in practically relevant primary cell types. More recent advances have, however, demonstrated freezing and good recovery of primary cells under small volumes of liquid cryoprotectant. Eskandari et al. [14]. were able to successfully recover porcine endothelial corneal cells by selecting a monolayer substrate with similar thermal expansion properties to that of ice. Also, Töpfer et al. [40] demonstrated cryopreservation of bovine colonic cell in 3-D organoid format within 96-well plates was successful in terms of post thaw viability and cytotoxic response compared to a control. The influence of ice nucleation temperature in these studies received relatively little attention and has not been investigated at all in the case of primary cells. A possible reason for this is the practical difficulty of simultaneously inducing ice nucleation in each and every well of a 96-plate at a discrete temperature in a way that does not disturb or contaminate the cells within. Campbell et al. [9] studied the effect of ice nucleation control during the cryopreservation of plated rat aorta and bovine corneal cell lines by separately using Snomax® (a commercial ice nucleating agent made from non-viable *Pseudomonas syringae* bacteria) and also a cryogenically cooled manifold device to control ice nucleation. While they saw some evidence of improvement in both the post-thaw cell viability and attachment rates when ice nucleation was controlled they were unable to induce ice nucleation across plates in a sufficiently uniform manner using these techniques.

Here we demonstrate, using cultures of primary bovine granulosa cells and a non-invasive method of inducing ice nucleation, that active control of the ice nucleation step is required for both successful and consistent cryopreservation of monolayers of primary mammalian cells in generic polypropylene 96-well plates. Granulosa cells surround and support oocyte growth and development in mammalian ovarian follicles, and they are the subject of intense research in their own right [35,44]. We used granulosa cells as a convenient primary cell model as these cells can be rapidly harvested from abattoir-derived ovarian tissues without the need for any enzymatic digestion allowing us to demonstrate proof of concept of the efficacy of our approach for in-plate somatic cell cryopreservation. We show that inducing ice nucleation in individual wells at high supercooled temperatures (less than 5 °C of supercooling) is vital for achieving good levels of post-thaw cell viability. Since our hypothesis is that the degree of supercooling is very important for high post-thaw cell recovery, we have gone to some effort to characterise freezing temperatures when ice nucleation is controlled and uncontrolled. We then discuss the reasons for this by reviewing observations of the supercooling behaviour of purified water over a wide range of aliquot volumes and why this has hindered the efficient cryopreservation of cells within 96-well plates from being conducted on a larger scale.

## 2 Materials and methods

### 2.1 Controlled and uncontrolled ice nucleation temperatures within 96-well plates

To quantify the variability of temperatures of controlled and uncontrolled freezing in the multiwell plates used in this study we have used the IR-NIPI (Infra-Red – Nucleation by Immersed Particle Instrument) to determine the range of ice nucleation temperatures that occur when sub-mL volumes of ultrapure water and of cryoprotectant solution are slow-cooled (1 °C min^−1^) to cryogenic temperatures. The IR-NIPI comprises a controlled rate freezer (Asymptote ViaFreeze Research) and an integrated IR camera (Fluke Ti9) which is used to observe the temperatures of individual wells within a multiwell plate mounted upon the cooling stage. This system avoids the use of thermocouples in direct contact with the liquid in the wells which may themselves trigger nucleation. Temperatures of individual wells are logged every 15 seconds using the IR camera and nucleation events are captured by the rapid release of latent heat which occurs when the contents of the wells freeze. The ice nucleation temperature for a well is taken as the temperature recorded immediately before a sharp temperature increase and subsequent stabilisation at the expected melting point of the liquid. A full description of the IR-NIPI apparatus is detailed in Harrison et al. [17].

### 2.2 Bovine granulosa cell cultures

Bovine granulosa cells were cultured after Wrathall and Knight [44] and Picton et al. [35]. Bovine ovaries were transported to the laboratory at ambient temperature from the local abattoir (JC Penny and Sons, Rawdon, Leeds, UK), and were cut from the reproductive tract as received and washed very briefly in 70% (v/v) ethanol to remove unwanted detritus. The ovaries were then decanted and washed in room temperature phosphate buffered saline (PBS) containing 100 kIU L^−1^ penicillin and 0.1 μg L^−1^ streptomycin at 39 °C. Granulosa cells were extracted from the ovaries by needle aspiration from individual follicles using a 10 mL syringe fitted with a 19G needle. The follicle aspiration media (AM) was made up of M199 media containing 10 mM HEPES, 100 kIU L^−1^ penicillin, 0.1 μg L^−1^ streptomycin, 1 mg L^−1^ amphotericin and 3000 IU heparin L^−1^, pre-equilibrated to 39 °C. A small amount of media was taken up into the syringe before the follicles were gently punctured and aspirated up and down after gently scraping the walls of the follicles to dislodge the granulosa cells. Cells were then decanted into a 50 mL polypropylene tube. This process was repeated with multiple ovaries (typically 5 to 8) to obtain a stock with a sufficient number of cells to seed a set of plates for each freezing experiment. When cell extraction was complete the cells were washed by centrifugation for 10 min at 250 × *g* at room temperature. After washing the cells were re-suspended in 5 mL of pre-equilibrated culture media (CM) at 39 °C, consisting of McCoy's 5a medium supplemented with 20 mM HEPES, 100 kIU L^−1^ penicillin, 0.1 μg L^−1^ streptomycin, 3 mM L^−1^ l-glutamine, 10 μg L^−1^ bovine insulin, 2.5 mg L^−1^ transferrin, 4 μg L^−1^ sodium selenite and 10% v/v foetal calf serum. In order to break up cell aggregates the cells were repeatedly gently aspirated through two stacked pipette tips. The cell stock concentration was determined by trypan blue dye exclusion and counting with a haemocytometer. The stock was subsequently diluted with CM to achieve a plating density of 10^5^ viable cells per well in 200 μL of CM in sterile polypropylene flat bottom 96-well plates (Thermo Scientific Nunclon™ Delta Surface, Cat. No. 167008). Cells were cultured for 72 h at 39 °C in a 5% CO_2_ humidified atmosphere with complete media changes conducted after 48 h of culture. All media and additives were purchased from Sigma-Aldrich (Poole, Dorset, UK).

### 2.3 Cryopreservation of 96-well plates

**Fig. 1:**
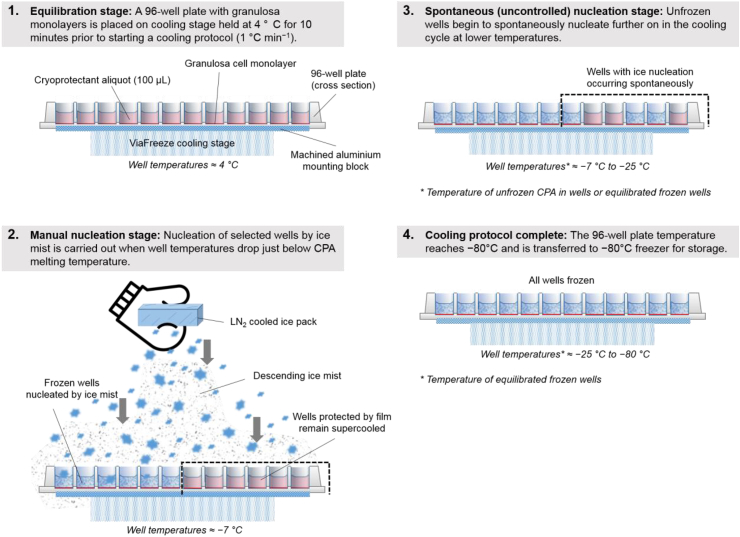
Description of 96-plate cryopreservation procedure and manual ice nucleation technique.

After culturing for 72 h the granulosa cells in the 96-well plates consistently formed confluent fibroblastic monolayers. To prepare for freezing, the 96-well plates with confluent cells were cooled to approximately 4 °C by placing them on an ice-cooled aluminium mounting block specially machined for maximum thermal contact with the underside of the plate. Once equilibrated the CM was removed from each well and replaced with 100 μL of cryoprotectant (CPA) media consisting of CM with 10% (v/v) dimethyl sulfoxide and 0.1 M trehalose. The CPA was chilled to 4 °C in a refrigerator prior to use to minimise toxicity and after addition of CPA the plates were left to equilibrate at 4 °C for 10 min. Then the plates along with mounting block were placed on the cooling stage of a controlled rate freezer (Asymptote ViaFreeze Duo, Asymptote Ltd, Cambridge, UK) and a protocol was initiated which cooled the plates from 4 °C to −80 °C at a rate of −1 °C min^−1^. Ice nucleation treatments were applied and once the cooling protocol was complete plates were transferred immediately to a −80 °C chest freezer for storage. An illustration of the controlled rate freezing process and ice nucleation steps is provided in Fig. 1.

### 2.4 Manual induction of ice nucleation in 96-well plates

Ice nucleation was simultaneously induced by manual nucleation and left uncontrolled to occur spontaneously in subsets of wells within the same plate undergoing controlled rate cooling. Manual ice nucleation was achieved by exposing wells to ice mist descending from a liquid nitrogen-cooled ice pack held about 10–15 cm above the plate (Fig. 1, panel 2). Ice crystals, formed as moisture in the cooled air around the cold object condensed and froze, acted as seed crystals as they fell into the supercooled CPA in the exposed wells. This caused nucleation to occur across all wells of the plate both in a very narrow range of temperatures and within 5 °C of the melting point of the CPA. The well nucleation temperatures using this method and CPA melting temperature were determined by infra-red thermometry to be approximately −7 °C ± 1 °C (*n* = 24) and −4 °C ± 1 °C (*n* = 15) respectively. This method of manual nucleation is less invasive than a previously reported mechanical protocol used for 96-plate freezing which required physical intervention [9]. Where ice nucleation was intended to occur uncontrolled during cooling, subsets of wells were covered with laboratory film to prevent exposure to the falling ice mist during manual nucleation (Fig. 1, panels 2 and 3). Ice nucleation within wells was visually confirmed by a sudden change of appearance of the well liquid from clear to translucent. This was consistently observed at a much later time points (and correspondingly lower temperatures) during the cooling protocol in the spontaneously nucleating wells qualitatively confirming the greater degree of supercooling in these wells. Further details of well freezing temperature measurement is provided in Section 3.1.

### 2.5 Thawing of cryopreserved 96-well plates

After 3–4 days of storage, 96-well plates were removed from the −80 °C freezer and placed into an incubator held at a temperature of 39 °C. Aluminium plates shaped to fit to the top and bottom of the 96-well plate and pre-heated to a temperature of 39 °C were attached to either side of the plate in the incubator. After 10 min the plates were removed from the incubator and the CPA media was removed as rapidly and thoroughly as possible with a multichannel micropipette. 200 μL of CM containing 50 μg mL^−1^ vital dye neutral red was then added to each well and the plates placed in the incubator at 39 °C in a 5% CO_2_ atmosphere for 3 h. At this point the dye uptake was complete and the plate was ready for viable cell counting.

### 2.6 Neutral red assessment of cell number and viability

A viable cell counting assay based on the uptake of neutral red by only living cells [5] allowed rapid assessment of the number of live granulosa cells present in individual wells and was used to determine viable cell numbers per well of cryopreserved plates upon thawing and also of non-frozen control plates. The 96-well plates containing granulosa cultures were, as mentioned above, incubated for 3 h in 50 μg mL^−1^ neutral red dye in CM. 200 μL of a washing solution (WS) containing 4% formaldehyde and 1% CaCl_2_ in distilled water was then added to each well and left to stand for 3 min. The wash solution was then carefully removed and 200 μL of fixing solution (FS) containing 1% (v/v) glacial acetic acid and 50% (v/v) ethanol was added to release the dye retained by viable cells. The plates were then left to stand for 30 min before the absorbance of each well (dye concentration) was measured at 540 nm using a microplate reader (Thermo Scientific Multiskan GO) with analysis software (Thermo Scientific SkanIt™ Software v. 4.1). To translate dye concentration into viable cell number per well in the frozen-thawed plates the absorbance of cells in test wells were compared to a standard curve. The standard curve was derived from a dilution series of a known quantity of neutral red stained and lysed granulosa cells (typically in the range of 4 × 10^3^ to 4 × 10^5^ cells per well) and measured in triplicate at 540 nm.

### 2.7 Assessment of cell morphology

The visual appearance of the cell monolayers *in vitro* was recorded before and after freezing using a Nikon Eclipse Ti inverted microscope at 250x magnification fitted with a digital camera and processed with RI Viewer Imagining Software. The cells were incubated with neutral red dye before imaging as previously described above.

### 2.8 Cryoprotectant toxicity testing

In order to determine the cytotoxic impact of the CPA on the granulosa cells separately from the impact of freezing, 100 μL of CPA at 4 °C was added to plates containing granulosa at the 72 h time point and left in contact for a time period identical to the cell exposures used during the cryopreservation protocol. The CPA was then removed with a multichannel micropipette, replaced with the CM-neutral red solution before returning to the incubator for 3 h. Finally viable cell number in the plate was quantified using the using the neutral red assay as detailed above.

### 2.9 Experimental design and statistical analysis of post-thaw cell viability

Eight independent replicate bovine granulosa cell cultures (*a-h*) were conducted and each were seeded into separate 96-well plates. In each plate equal subsets were designated for a range of ‘treatments’ after culturing. These comprised a subset not subject to any addition of CPA or freezing treatment and used as a control (‘Baseline’); a subset subject to addition of CPA and cryopreservation using manually controlled nucleation (‘Controlled Nucleation’); a subset subject to addition of CPA and cryopreservation with uncontrolled ice nucleation (‘Uncontrolled Nucleation’); and a subset subject to addition of CPA without freezing as an indicator of CPA toxicity (‘CPA Treatment Only’).

A two-sample T-test of the viable cell number means of Baseline and CPA Treatment only tests from all 8 culture replicates showed no significant difference between the groups (*p* = 0.835). This confirmed that the addition of CPA *per se* had no significant toxic impact on the viability of cultured granulosa cells, accordingly this variable was disregarded during the statistical comparisons of the Baseline, Controlled Nucleation and Uncontrolled Nucleation data subsets. The differences in mean viable cell numbers between the Baseline, Controlled Nucleation and Uncontrolled Nucleation wells for each culture replicate were analysed by Welch's one-way ANOVA with a Games-Howell post-hoc test to determine differences of means. These tests were selected as they assumed non-equivalent variances between the groups which arises from the fact that uncontrolled nucleation results in a very wide range of freezing temperatures compared with that of the manual nucleation method. Finally to establish relative cell viability for each group (i.e. % survival) after cryopreservation the mean cell number for the treated plates was divided by the cell number measured in the Baseline (unfrozen) plate.

## 3 Results

### 3.1 Freezing temperatures with controlled and uncontrolled nucleation

**Fig. 2:**
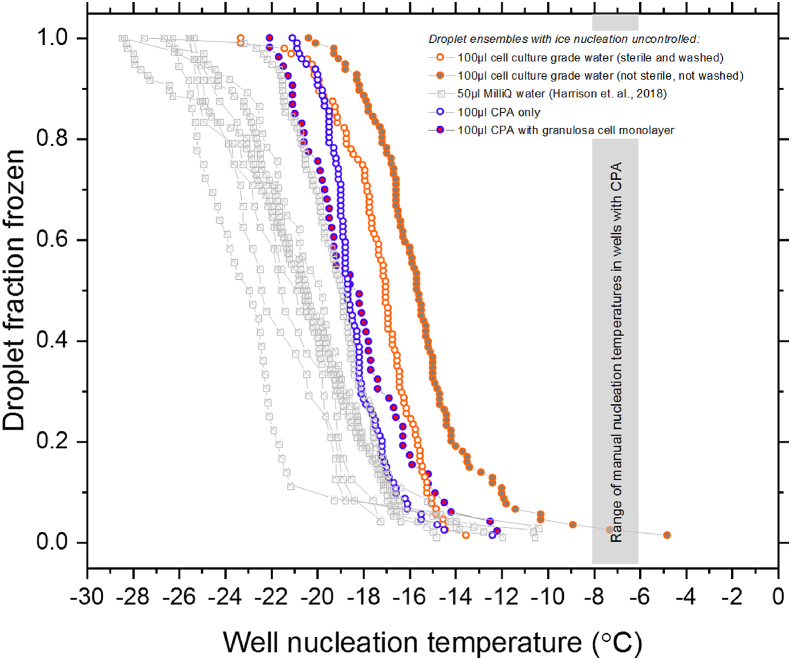
Droplet fraction frozen against well ice nucleation temperature for ensembles of 100 μL droplets of purified water prepared in different conditions of sterility and of CPA with and without a cell monolayer in polypropylene 96-well plates. This variability of freezing temperature is representative of uncontrolled nucleation while the expected range of freezing temperatures induced by ice-mist manual nucleation method is depicted for reference. The temperature uncertainty of the IR-NIPI used is ±0.9 °C.

The freezing temperatures where ice nucleation was not controlled for 100 μL volumes of cell culture grade water (Hyclone HyPure, GE Healthcare Life Sciences) and CPA in 96-well plates from the IR-NIPI instrument alongside previous data for 50 μL MilliQ water are shown in Fig. 2. Generally freezing occurred both at much lower temperatures and over a wider range of temperatures when nucleation was uncontrolled compared with when it was controlled with the ice mist method. Also, the freezing temperature of the 100 μL volumes was generally warmer than the freezing temperatures in the 50 μL volumes; this was expected, since the probability of freezing generally increases with volume [4]. The plates containing water were prepared either aseptically in a laminar flow hood with a washing step (rinsing with cell culture grade water) or simply in an ambient laboratory setting without any washing to determine the effect of potential airborne contamination on freezing temperatures. This tentatively showed that the effect of sterile preparation and washing was to lower the variability and slightly lower the overall freezing temperatures. Nevertheless, the freezing temperatures were both generally much lower and far more variable than those which result from manual nucleation. The freezing temperatures for the CPA loaded plates show a comparable level of variability while median nucleation temperatures are about 4 °C lower. This would be expected due to the colligative effect of solutes on heterogeneous ice nucleation. Tests with CPA in the wells, with and without granulosa cells showed that the presence of a cell monolayer had no apparent effect on freezing temperatures.

**Fig. 3:**
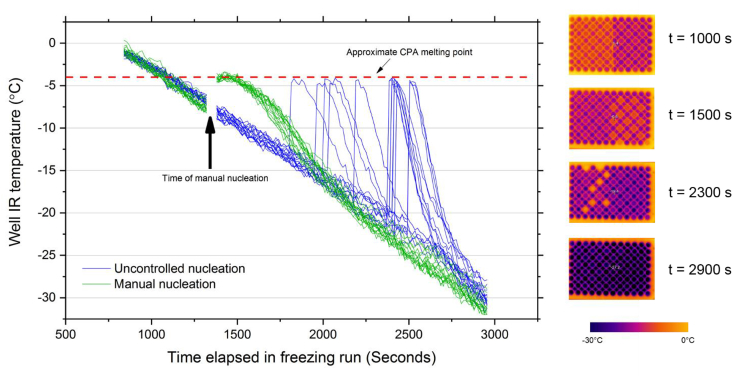
**(Left):** IR-NIPI temperature log of 96-well plate loaded with 50 μL aliquots of CPA where half of the wells were nucleated manually with ice mist (green lines) and half left to nucleate without control (blue lines). The data gap at around 1300 s is due to the apparatus being disabled to allow the manual nucleation procedure. **Right:** IR colour map mages of the plate undergoing cooling at progressing time points – manually nucleated wells are on the right half of the plate, uncontrolled on the left. Recently frozen wells having released latent heat appear yellow (temperature-colour scale below is approximate). A plastic film covering the left side of the plate appears as an orange region in the image taken at 1000s. This was placed to protect the wells on this side of the plate from the nucleation inducing ice mist. After manual nucleation was done the film was removed so is no longer visible by the next image at 1500 s. (For interpretation of the references to colour in this figure legend, the reader is referred to the Web version of this article.)

We also used the IR-NIPI instrument to record the temperature of each well during cooling in order to quantify the temperature profile of the wells as they heated up during freezing and then cooled down again after freezing (see Fig. 3). This was done by loading a 96-plate with 50 μL aliquots of CPA, where we controlled ice nucleation for half of the plate and left it uncontrolled in the other half (n = 15 wells for each group). Alternate wells were left empty to avoid artefacts caused by latent heat release being transferred to adjacent wells upon freezing. Fig. 3 shows that controlling nucleation manually resulted in a very narrow range of nucleation temperatures all at higher temperatures (approximately −7.0 °C; a supercooling of about 3 °C) compared with those wells where nucleation occurred uncontrolled (−12.9 °C to −22.5 °C, median −20.5 °C). As the manual nucleation procedure required temporary disabling of the IR camera for roughly 45 seconds it was not possible to observe well temperatures at the exact point of nucleation. However, nucleation was visually observed to be uniform and instantaneous across these wells and any variation in nucleation temperatures across this group was probably due to a small (±1 °C) cross-plate temperature gradient. Also apparent from Fig. 3 is that uncontrolled nucleation results in much greater cooling rates after nucleation (up to 10.3 °C min^−1^), whilst those wells nucleated manually displayed a cooling rate of up to 3.5 °C min^−1^, much closer to the nominal plate cooling rate of 1 °C min^−1^. A more modest cooling rate is thought to be advantageous for cell survival, hence this is another reason for controlling ice nucleation.

Overall, these tests show that controlling freezing with an ice mist dramatically reduces the supercooling experienced by the cells, reduces the variability in supercooling and also reduces the cooling rate that they experience.

### 3.2 The effect of controlling nucleation on post thaw cell viability

**Fig. 4:**
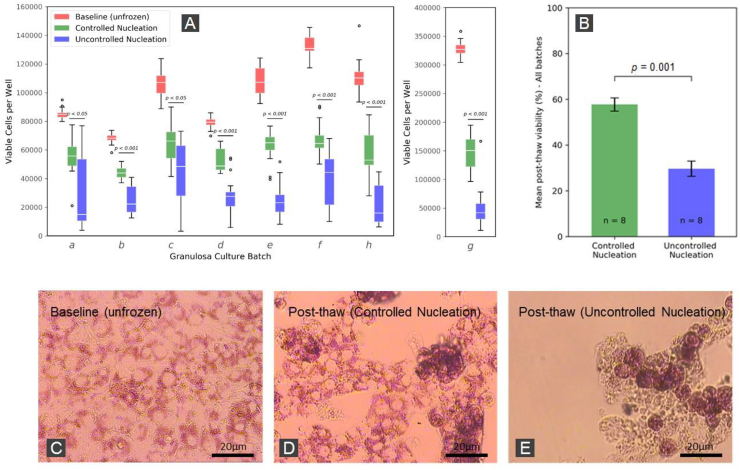
**(A):** Boxplots of viable granulosa cell number pre- and post-cryopreservation as determined by Neutral Red assays. All treatments within all groups are shown to be significantly distinct by ANOVA post-hoc tests with results of post-hoc analyses between Controlled and Uncontrolled Nucleation treatments for each cell batch shown as *p* values. Batch g is shown in separate pane with its own y-axis because the cell density was 2–3 times higher than the other batches. n = 24 for each Baseline group and n = 16 for each Controlled Nucleation and Uncontrolled Nucleation group. **(B):** Cell viability post-thawing relative to Baseline averaged across all cell batches with result of two sample *t*-test. Values plotted are means ± SEM for the number of replicates shown. **(C**–**E):** Micrographs of adhered bovine granulsoa cells stained with Neutral Red dye. Panel C shows cells in the non-cyropreserved control plate at the end of the 72 h incubation period. Panels D and E depict representive morphologies of thawed cells immediately after cryopreservation with and without ice nucleation control respectively. (For interpretation of the references to colour in this figure legend, the reader is referred to the Web version of this article.)

The number of viable cells per well determined by the Neutral Red assay for the Baseline and post-freezing treatments for culture replicates *a-h* are presented in the box plots in Fig. 4A. Qualitatively, there is clearly a trend, with the number of viable cells being generally higher when nucleation was controlled than when it was uncontrolled. However, there is also a great deal of variability, both across plates and batches. ANOVA analysis found the differences between mean cell viability for each treatment (controlled and uncontrolled ice nucleation) are significant (*p* < 0.01) across all granulosa culture batches. Furthermore post-hoc analysis between the Controlled and Uncontrolled Nucleation treatments showed that the Controlled Nucleation treatment resulted in significantly higher post-thaw viabilities in the case of all batches (*p* < 0.05 for batches *a* and *c; p < 0.001 for all remaining batches*).

The overall survival rates for Controlled and Uncontrolled Nucleation are shown in Fig. 4B. Across all batches a mean post-thaw recovery rate of 57.7% (Standard error of mean (SEM) = 8.3%) was observed when ice nucleation was controlled compared to a mean post-thaw recovery of 29.6% (SEM = 9.3%) when ice nucleation was left uncontrolled. A 2-sample T-test found the difference of 28.1% to be statistically significant (*p* < 0.001) (Fig. 4B). It was therefore evident that controlling and restricting ice nucleation to high temperatures (1 °C ± 1 °C of supercooling) during controlled rate freezing of 96-well plates significantly increased the proportion of bovine granulosa cells which survived cryopreservation.

### 3.3 Effect of cryopreservation on cell morphology

Micrographs shown in Fig. 4C–E contrast the representative morphologies of the granulosa cells before and after cryopreservation and thawing, illustrating the effects of ice nucleation control. Non-frozen cells (Fig. 4C) show a homogenously adherent monolayer whilst thawed cells (Fig. 4D and E) are more clumped and appear attached to the well bottom by cellular processes. The viable (indicated by red staining) adherent cells are, however, more abundant in the manually nucleated plate (Fig. 4D) compared to the uncontrolled nucleation plate (Fig. 4E) where several non-viable, unstained cells are visible. A loss of cell layer coverage indicative of post-thaw detachment is also apparent in the frozen plates, the degree of which appeared most severe in the uncontrolled plate (Fig. 4E). These observations are qualitatively consistent with the viability analysis detailed in the previous section.

## 4 Discussion

In our experiments controlling ice nucleation by seeding with an ice mist improved post-thaw cell viability from only 29.6% when ice nucleation was uncontrolled to 57.7% (*p* < 0.001). These cell viability results are consistent with previous work which demonstrates that induction of ice nucleation at high temperatures improves cryopreservation results [6,20,22,23,25,30,34,43] and are consistent with the classic two-factor picture of freezing injury (expanded on below) [28,30]. The poor post-thaw cell viability when ice nucleation is uncontrolled is most likely related to the high degree, and variable nature, of supercooling of the small aliquot volumes (100s of μL) typically used in 96-well plates. This unpredictable variability in well ice nucleation temperatures compared with where ice nucleation was controlled was confirmed by our IR thermometry measurements in addition to much more severe temperature fluctuations.

The enhanced cell survival with controlled freezing can be rationalised through an understanding of the fundamental processes that occur in the freezing process. For example, one factor mitigated by reducing the degree of supercooling is the rapid cooling after ice crystal growth experienced by cells when freezing occurs at deep supercooling (see Fig. 3). Also, water diffusion is slowed by increasing the component of initially formed ice volume as a result of more supercooling [30,42]. This, coupled with faster than intended cooling rates, can result in cells not being able to dehydrate quickly enough and increasing the chance of lethal intracellular ice formation. Furthermore cells frozen in monolayer form have previously been found to have a higher susceptibility to intracellular ice formation compared to equivalent cells in suspension, this attributed to intercellular ice formation travelling from cell to cell through the monolayer [1]. Further work is needed to supplement findings on immediate post-thaw viability and confirm that granulosa cell functionality is retained post thaw. This could be done, for example, by extended culture and measurement of specific granulosa cell steroidogenic function [35].

We sometimes observed that adherent cells would detach from the base of the wells upon thawing. This observation is consistent with previous work by others [9,12,13] attempting to cryopreserve cell monolayers either in multiwell plates or on other substrates. However from visual inspection the degree of detachment was found to be more severe in wells which experienced deeper supercooling which has not previously been reported. The cells that detached in our experiments may have simply been the ones that did not survive the freezing and/or thawing processes. Adherent cells, however, having become rigid at cryogenic temperatures may have also become mechanically separated from the substrate during the freezing and thawing cycle despite remaining viable [13].

Campbell et al. [7] attributed cell detachment to thermal expansion of the plate when warmed too rapidly. Having warmed all our plates by the same method and therefore at the same rate we cannot solely attribute the degree of detachment to warming rate. Moreover, Eskandari et al. [14] and Rutt et al. [38] recently tackled the mechanical detachment of cell monolayers by freezing cells on substrate with a similar coefficient of linear expansion to that of ice. They hypothesised that eliminating the differential expansion of ice and substrate prevents buckling or splitting of the cell monolayer over the course of a cooling and thawing cycle culminating in detachment. In our case as we used the same plate type for each freezing run we did not vary the ice-substrate thermal mismatch. However controlling (increasing) the ice nucleation temperatures did result in much less severe temperature fluctuations during the cooling cycle which may have led to less severe thermal stresses, thus protecting the integrity of the monolayer.

Detachment of cells from the substrate post thaw presents a technical obstacle for producing frozen plated cells on a commercial basis since detached cells would be lost during the CPA removal and washing steps. These observations of cell detachment in our thawed granulosa cultures adds the importance of controlling ice nucleation to the list of favourable factors identified by previous workers, such as substrate composition and controlled warming, for plated cell monolayer cryopreservation protocols. Further work quantifying the degree of cell detachment, viability of detached cells and trialling plates with different compositions would confirm this.

**Fig. 5:**
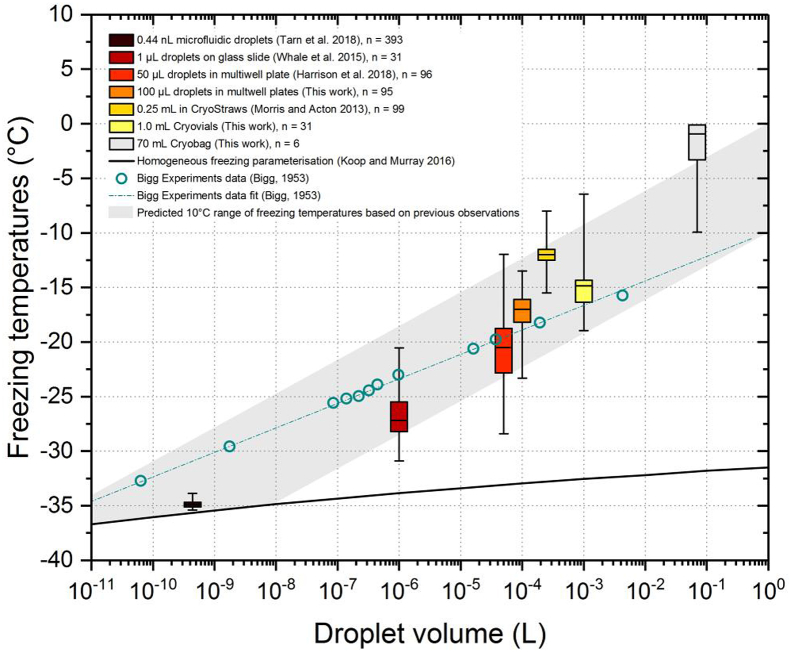
Freezing temperatures of purified water droplet ensembles at a range of volumes with an empirical trend. Boxes denote 25th – 75th percentiles, bars denote medians and whiskers denote range of values. We have plotted literature data [4,5,13,25,34,37] of ice nucleation temperatures of pure water aliquots in various containers and spanning volumes from sub-pL to tens of mL along with a homogenous freezing parameterisation [16]. We also show new data for 100 μL droplets in a 96-well plate (from Fig. 2) and data for 1.0 mL in cryovials (Corning 2.0 mL capacity, Cat No. 430488) and 70 mL in cryobags (Milenyi CD250). We used Hyclone cell culture water. The cryovials assay was performed using the IR-NIPI as previously described (n = 31); while for the cryobags the ice nucleation temperatures (n = 6) were determined using a thermocouple as the bags were cooled while inside aluminium cassettes.

Since ice nucleation temperature is clearly an important consideration in the cryopreservation of cells, we will now discuss freezing in typical cryopreservation containers with a range of volumes. We summarise literature data and some new data for pure water freezing temperatures for vessels with a range of volumes in Fig. 5. Truly pure liquid water, containing no contaminating particles and in contact with no surface, can nucleate via homogeneous nucleation which can be described with Classical Nucleation Theory (CNT) [21]. Homogeneous ice nucleation is practically possible to achieve only in very small droplets and is readily accessed in droplets of around a nanolitre in volume or smaller [2,39]. However all the freezing events in vessels of volumes relevant to cryopreservation applications (multiwell plates, vials, straws etc.) tend to occur at temperatures clearly too warm to be homogeneous, yet apparently occur ‘randomly’ within a predicable range of values correlated with the aliquot volume (Fig. 5). Stringent efforts to eradicate heterogeneous ice nucleating contaminants in the liquids or ambient atmosphere by washing and preparing in a laminar flow hood made only a minor difference to the well freezing temperatures. Nevertheless it is apparent a larger aliquot volume or substrate surface area increases the chances of one of these sites being present. Indeed, successful cryopreservation in large volumes such as cryobags may not require control of ice nucleation because they freeze spontaneously anyway at these high temperatures. In contrast, multiwell plates containing 10s of μLs are highly susceptible to supercooling. Hence, in order to achieve successful cryopreservation in multiwell plates, the control of ice nucleation is needed.

## 5 Conclusions

We have demonstrated that primary bovine granulosa cells can be successfully cryopreserved in monolayers in a generic 96-well culture plate format using controlled-rate freezing with a non-invasive manual ice nucleation technique. Using a remote IR thermometry technique we quantified, for the first time remotely, the supercooling behaviour of aliquots of cryoprotectant in a 96-well plate. The granulosa cell culture freezing survival rates coupled with the IR measurement of freezing temperatures in multiwell plates indicate a first order link between higher ice nucleation temperatures and better rates of post-thaw survival. We also describe for the first time a qualitative link between higher ice nucleation temperatures and a reduction in the degree of post-thaw cell detachment which is known to be a problem when freezing cells in monolayer form. Optimal conditions such as cryoprotectant formulation, thermal expansion properties of the substrate, cooling rate and thawing method are likely to differ when cryopreserving differing cell types in multiwell plates. Controlling ice nucleation at high temperatures should now also be considered as standard protocol as we also have shown that deep and variable supercooling occurs when sub-mL aliquots of cryoprotectant are cooled to cryogenic temperatures in 96-well plates. This finding should prove useful for the development of high throughput screening of cryobiological procedures, facilitating quick testing of new cryoprotectants and treatments in a primary cell system. Additionally, there are applications where cryopreservation of cells directly in 96-well plates will be of use, notably testing of drug molecules on, for example, primary hepatocytes and cardiomyocytes. This work indicates that control of ice nucleation will likely be critical for the success of any such application. Therefore further work is proposed to cryopreserve a wider range cell types, expand post-thaw performance metrics to cell functionality as well as viability and assess methods of controlling ice nucleation in 96-well plates in a potentially cGMP compliant manner.

## Data Availibility

The dataset for this paper is publicly available at the University of Leeds Data Repository - https://doi.org/10.5518/782 (Daily et al., 2020).
